# Estimation of Wheat Agronomic Parameters using New Spectral Indices

**DOI:** 10.1371/journal.pone.0072736

**Published:** 2013-08-30

**Authors:** Xiu-liang Jin, Wan-ying Diao, Chun-hua Xiao, Fang-yong Wang, Bing Chen, Ke-ru Wang, Shao-kun Li

**Affiliations:** 1 Institute of Crop Science, Chinese Academy of Agricultural Sciences/Key Laboratory of Crop Physiology and Production Ministry of Agriculture, Beijing, China; 2 Beijing Research Center for Information Technology in Agriculture, Beijing, China; 3 Key Laboratory of Oasis Ecology Agriculture of Xinjiang Construction Crops, Shihezi, China; 4 Institute of Cotton, Xinjiang Academy of Agricultural Reclamation Sciences, Shihezi, China; University of Nottingham, United Kingdom

## Abstract

Crop agronomic parameters (leaf area index (LAI), nitrogen (N) uptake, total chlorophyll (Chl) content ) are very important for the prediction of crop growth. The objective of this experiment was to investigate whether the wheat LAI, N uptake, and total Chl content could be accurately predicted using spectral indices collected at different stages of wheat growth. Firstly, the product of the optimized soil-adjusted vegetation index and wheat biomass dry weight (OSAVI×BDW) were used to estimate LAI, N uptake, and total Chl content; secondly, BDW was replaced by spectral indices to establish new spectral indices (OSAVI×OSAVI, OSAVI×SIPI, OSAVI×CI_red edge_, OSAVI×CI_green mode_ and OSAVI×EVI2); finally, we used the new spectral indices for estimating LAI, N uptake, and total Chl content. The results showed that the new spectral indices could be used to accurately estimate LAI, N uptake, and total Chl content. The highest R^2^ and the lowest RMSEs were 0.711 and 0.78 (OSAVI×EVI2), 0.785 and 3.98 g/m^2^ (OSAVI×CI_red edge_) and 0.846 and 0.65 g/m^2^ (OSAVI×CI_red edge_) for LAI, nitrogen uptake and total Chl content, respectively. The new spectral indices performed better than the OSAVI alone, and the problems of a lack of sensitivity at earlier growth stages and saturation at later growth stages, which are typically associated with the OSAVI, were improved. The overall results indicated that this new spectral indices provided the best approximation for the estimation of agronomic indices for all growth stages of wheat.

## Introduction

The development of remote sensing has provided opportunities to quantitatively describe agronomic parameter changes across all growth stages of crops. The application of remote sensing to agronomic problems has created new methods to effectively improve field crop management. Many authors have provided detailed information about the relationships between spectral indices and agronomic parameters, including the leaf area index (LAI), nitrogen (N) uptake, total chlorophyll (Chl) content, and so on.

The LAI is a key variable for the diagnosis and prediction of crop growth and yield. This makes the LAI critical for effective understanding of the biophysical processes of plant canopies and the prediction of plant growth and productivity [1]–[6]. Rouse et al. suggested that the most well-known and widely used vegetation index was the normalized difference vegetation index (NDVI), and found that NDVI was linearly correlated with the leaf area index (LAI) in crop fields [7]. However, the NDVI does possess certain limitations related to soil background brightness, in that the NDVI tends to be affected by different soil color and moisture conditions [8]–[10]. To overcome this problem, Rondeaux et al. proposed using an optimized soil-adjustment factor, and obtained an optimized soil-adjusted vegetation index (OSAVI), which mitigated the effects of soil and moisture conditions [11].

Nitrogen is a critically important element that is monitored in an effort to maintain crop health, while there is a good relationship between nitrogen content and chlorophyll content [12]–[13], therefore chlorophyll content is an important indicator for nitrogen fertilizer applications. Stone et al. detected and predicted N uptake in winter wheat using hand-held sensors [14]–[15]. Osborne et al. identified the important reflectance wavelengths for the prediction of N concentration changes at different growth stages [16]. Gitelson et al. suggested that the green NDVI (GNDVI) was more sensitive than the NDVI for wheat N uptake over 100 kg/ha [17], but Moges et al. indicated that the NDVI was more robust than the GNDVI for prediction of crop N uptake [18]. Certain researchers have proposed many N indicators for the assessment of crop N change according to the spectral features of chlorophyll in the visible and red-edge bands [19]–[20]. A good relationship between a combined index (the ratio of modified chlorophyll absorption ratio index and modified triangular vegetation index2, MCARI/MTVI2) and leaf nitrogen concentration was found by Eitel et al. [21]. Fitzgerald et al. reported that a spectral index, the canopy chlorophyll content index (CCCI), could predict canopy N, and specifically found a good relationship between the CCCI and canopy N [22]. Vigneaua et al. used field hyperspectral imaging as a non-destructive method to assess leaf nitrogen content in wheat [23]. And recently, remote sensing methods have been developed and applied for the prediction of Chl content for field crop management [24]–[30]. Gitelson et al. identified the best vegetation indices for the estimation of Chl content [30]. A good correlation between total Chl content and (R_NIR_ which is in the near infrared band reflectance/R_red edge_ which is in the red edge position reflectance)–1, (R_NIR_/R_green_ which is in the green band reflectance)–1 was found by Gitelson et al. [31], and Gitelson et al. applied the new vegetation index (R_NIR_/R_red edge_–1 and R_NIR_/R_green_–1) for the estimation of Chl content and indirectly estimated gross primary production (GPP) [32], [33].

However, spectral indices still eixst insensitivity at earlier growth stages, and saturation at later growth stages. Because biomass dry weight (BDW) was accumulated gradually as growth stages progressed, and insensitivity at earlier growth stages and saturation at later growth stages was not observed. Consequently, the objectives of this study were to (1) combine OSAVI and BDW for estimating LAI, N uptake, and total Chl content in wheat; (2) replace BDW with spectral indices; (3) and attempt to propose new spectral indices, in an effort to improve insensitivity at earlier growth stages and saturation at later growth stages.

## Materials and Methods

### 2.1. Design of Experiment

Field experiments were conducted in 2009 and 2010 at the ShiHezi University experiment site (44°20′N, 86°3′E), Xinjiang Province, China. The experiment site had representative soil types and crop management practices for Xinjiang Province, China. The soil was fine-loamy with a total N content of 42.6 mg kg^−1^, Olsen P of 26.5 mg kg^−1^, exchangeable K of 139.4 mg kg^−1^, and organic matter content of 11.6 g kg^−1^ in the 0–30 cm layer. Three local wheat cultivars: Xinchun 6, Xinchun 17, and Xinchun 22, were planted on April 5th 2008 and April 8th 2009. Nitrogen fertilizer as urea was applied at four rates (0, 105, 225, and 345 kg N ha^−1^) before planting. The N application was distributed at three stages in the growth process in the following percentages: 50% at seeding, 25% at jointing, and 25% at booting. For all treatments, 99 kg ha^−1^ P_2_O_5_ (as monocalcium phosphate [Ca(H_2_PO_4_)_2_]) and 150 kg ha^−1^ K_2_O (as KCl) was applied prior to seeding. The experiment was a 2-way factorial arrangement of treatments in a randomized complete block design with three replications for each treatment. Other management practices followed local standard wheat production practices.

### 2.2. Measurement of Canopy Reﬂectance

Spectral measurements were carried out at the following stages (2009, 2010): tillering (8th May, 6th May), jointing (20th May, 25th May), heading (8th June, 10th June), anthesis (18th June, 20th June), and filling (27th May, 25th May), respectively. All canopy spectral measurements were mounted on the tripod boom and held in a nadir orientation 1.0 m above the canopy. Measurements were taken under clear sky conditions between 10∶00 and 14∶00 (Beijing local time) using an ASD Field Spec Pro Spectrometer (Analytical Spectral Devices, Boulder, CO, USA). This spectrometer is fitted with a 25° field of view fiber optics, operating in the 350–2500 nm spectral region with a sampling interval of 1.4 nm between 350 and 1050 nm, and 2 nm between 1050 and 2500 nm, and with spectral resolution of 3 nm at 700 nm, and 10 nm at 1400 nm. A 40 cm × 40 cm BaSO4 calibration panel was used for calculating the black and baseline reﬂectance. To reduce the possible effect of sky and field conditions, spectral measurements were taken at four sites in each plot and were averaged to represent the canopy reflectance of each plot. Vegetation radiance measurement was taken by averaging 16 scans at an optimized integration time, with a dark current correction at every spectral measurement. A panel radiance measurement was taken before and after the vegetation measurement by two scans each time.

### 2.3. Agronomic Parameters Measurement

Immediately following spectral measurements, the leaf area index (LAI) was measured using the LAI-2000 Plant Canopy Analyzer (LI-COR Inc., Lincoln, NE, USA) with spectrometric measurements at the same position and gained by destructive sampling. The wheat was cut at the ground level and wet weights were recorded in 0.24 m^2^. Each plant was then dried at 70°C for 3 d and the dry weight was recorded. Dry plant material was then milled and analyzed in the laboratory. Total N content was estimated using a dry combustion method in a Dumas Elementary Analyser (Macro-N, Foss Heraeus, Hanau, Germany) [34].

The spectral measurements positions of the wheat leaves were collected using a hole puncher (diameter, 0.4 cm). Then, about 0.2 g wheat leaves of each sample was punched off in the laboratory. The selected samples were placed in 95% ethanol or acetone solution and allowed to stand for 24 hr in the dark. Following the 24-hr treatment, the leaves were white-green in color. Finally, leaf pigment density was measured using a colorimetric spectro-photometer. Absorbance of the supernatant was measured at 645, 652 and 663 nm, and chlorophyll a plus chlorophyll b content per unit leaf area was then calculated by the method of McKinney [35].

### 2.4. Selection of Spectral Indices

This study tested the spectral indices that were considered to be good candidates for estimation of plant chlorophyll content and LAI (Table 1) [11], [31], [36]–[37].

**Table 1:** Summary of spectral indices, wavebands and citations for LAI, nitrogen uptake and total chlorophyll content in this paper.

Index	Name	Formula	Developer(s)
OSAVI	Optimized soil-adjusted vegetation index	1.16×(R_800_−R_670_)/(R_800_+R_670_+0.16)	Rondeaux et al. (1996) [11]
SIPI	Structure insensitive pigment index	(R_800_−R_445_)/(R_800_−R_680_)	Penuelas et al. (1995) [36]
CI_red edge_	Red edge model	(R_800_/R_700_)−1	Gitelson et al. (2005) [31]
CI_green model_	Green model	(R_800_/R_550_)−1	Gitelson et al. (2005) [31]
EVI2	Enhanced vegetation index 2	2.5×(R_800_−R_660_ )/(1+R_800_+2.4×R_660_)	Jiang et al. (2008) [37]

Note: Ri denotes reﬂectance at band i (nanometer).

### 2.5. Statistical Analysis

Linear and nonlinear regression analysis was carried out using the biomass dry weight, OSAVI and OSAVI×biomass dry weight (the product of OSAVI and biomass dry weight) as independent variables, and the LAI, total Chl content, and N uptake (leaves yield N concentration of measured leaves) as dependent variables.

Statistically significant (p<0.05 or 0.01) coefficients of determination (R^2^) between three crop parameters (LAI, total Chl content, N uptake) and new vegetation indices or biomass dry weight were analyzed using SPSS software (16.0, SPSS, Chicago, IIIinois, USA). The R^2^ and root mean square error (RMSE) were used as metrics for quantifying the amount of variation explained by the relationships developed, as well as their accuracy. The performance of the model was evaluated through R^2^ and RMSE for the estimation of in-situ measured LAI, total Chl content and N uptake. Generally, the performance of the model was estimated by comparing the differences of the R^2^ and RMSE between the measured value and predicted value. The higher the R^2^ and the lower the RMSE were, the higher the precision and accuracy of model to predict agronomic parameters was considered to be.

## Results

### 3.1. Leaf Area Index (LAI)

**Figure 1:**
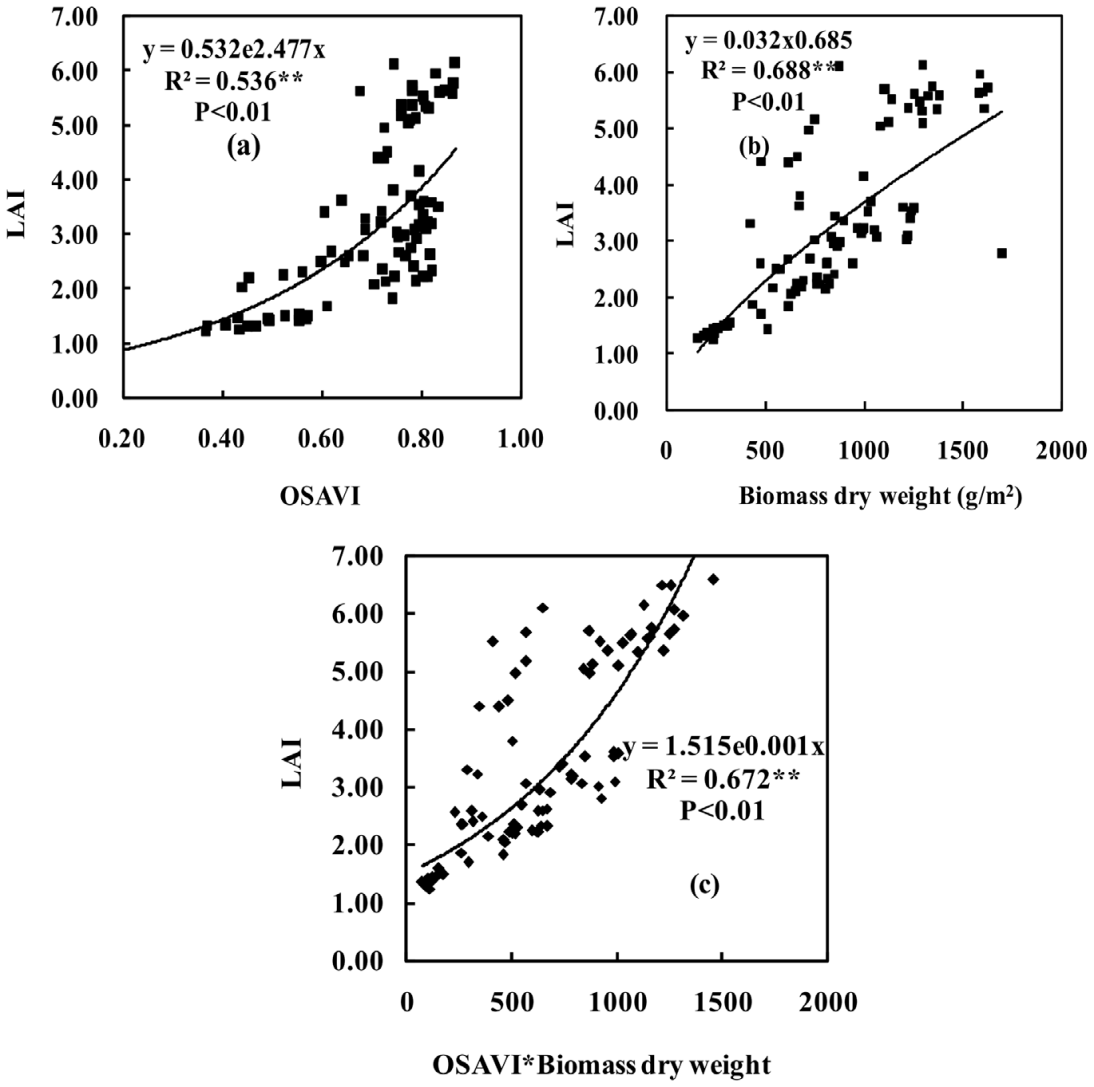
The coefficient of determination (R^2^) between OSAVI, biomass dry weight (BDW), BDW×OSAVI and LAI under the different nitrogen treatments for wheat (n = 90). Note: Probability levels are indicated by n.s., * and ** for ‘not significant’, 0.05, and 0.01, respectively.

A significant relationship was found between the OSAVI and LAI for the selected data, ranging from all growth stages of wheat across 2009 (Figure 1a). As noted above, the OSAVI was calculated and measured for each stage by averaging ASD-2500 sensor readings from taken at the vertical height of the canopy at 1.0 m. Steven showed that the LAI has a strong relationship with the OSAVI [38]. Figure 1a has shown that for the OSAVI index, there was a problem with saturation at later growth stages in wheat, determination coefficient (R^2^) value was 0.536. It is mainly for this reason that the OSAVI was not sensitive to the LAI (ranges from 4 to 6) at later growth stages. Possibly, this was because the band combination only considered the visible and near-infrared bands and was thus affected by environmental conditions.

Biomass dry weight and LAI were highly correlated (R^2^ = 0.688, *P<0.01*), independent of the area the wheat occupied. At earlier growth stages, the correlation between biomass dry weight and LAI (Figure 1b) was better than that between OSAVI and LAI (Figure 1a), R^2^ value was 0.688. The improvement was even greater at later growth stages. This is important because it indicates that biomass dry weight can be used to estimate wheat LAI, and to compensate for the OSAVI’s lack of sensitivity to the later growth stages changes in LAI. So, we used the OSAVI×biomass dry weight (BDW) index to estimate LAI changes using the data from all growth stages in wheat. The results showed that the OSAVI×BDW index was a good predictor of LAI, though the biomass dry weight alone was a more accurate predictor (Figure 1c). We performed non-linear regression on the OSAVI×BDW and LAI at all growth stages, and the resulting R^2^ was 0.672. These results suggested that the OSAVI×biomass dry weight index was an improvement over the OSAVI index, because the OSAVI alone was not sensitive to LAI changes at later growth stages.

### 3.2. Nitrogen Uptake

**Figure 2:**
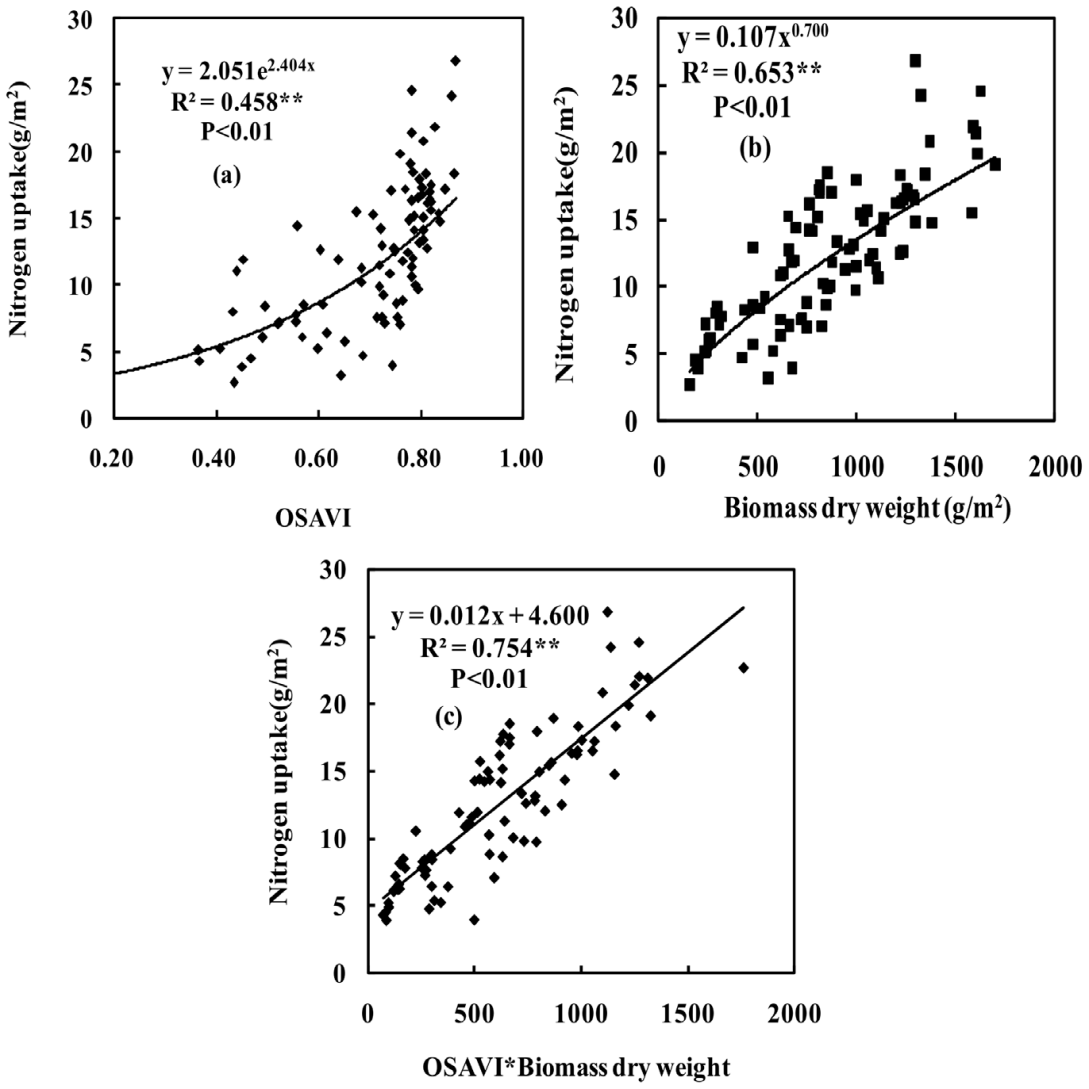
Relationship between OSAVI, biomass dry weight (BDW) and BDW×OSAVI and nitrogen uptake under the different nitrogen treatments for wheat (n = 90). Note: Same as above.

The amount of nitrogen (N) taken up in wheat was correlated with the OSAVI with an R^2^ value of 0.458 (Figure 2a). The results showed that the relationship between the OSAVI and N uptake was highly correlated (*P*<0.01) at all growth stages. However, at later growth stages when N uptake ranged from 15 to 27 g/m^2^, saturation was observed. A main reason for this problem was that the OSAVI was affected by other environmental factors [11]. Specifically, in this paper, the OSAVI saturation was mainly influenced by the structure of the wheat canopy. For example, the plant height of wheat canopy vary from 80 cm to 120 cm at the later growth stages, the reflectance at the top of wheat canopy (0–40 cm) can be detected by spectrometer (direct light), but the reflectance at the bottom of canopy (40–120 cm) cannot be detected by spectrometer (diffused light). The high-density wheat affected the spectral measurement and plant height. This caused the proportion of the diffused light to increase, thereby increasing the OSAVI saturation. The above problems led to a lack of sensitivity to the changes in N uptake at later growth stages. This suggested that with the increase in wheat biomass, the OSAVI became slightly affected by the surrounding environmental conditions, and the OSAVI and N uptake were correlated. The correlation between the OSAVI and N uptake could be explained by the ability of OSAVI to detect differences in red absorption. Moges et al. showed a similar finding, their research investigated the relationships of green, red, and near infrared bands to N uptake [16]. The results showed that the correlation between biomass dry weight and N uptake was better than that between OSAVI and N uptake, and the R^2^ value was 0.653 (Figure 2b).

The OSAVI×biomass dry weight index (BDW) could be used to estimate N uptake changes, and was a good predictor of N uptake. It performed better than either biomass dry weight or OSAVI alone in the prediction of N uptake for wheat. The results showed that a linear regression performed between the OSAVI×BDW and N uptake at all growth stages resulted in an R^2^ value of 0.754 (Figure 2c). This result suggested that the OSAVI×BDW index could be effectively used to improve the OSAVI’s prediction accuracy for N uptake.

### 3.3. Total Chlorophyll Content

**Figure 3:**
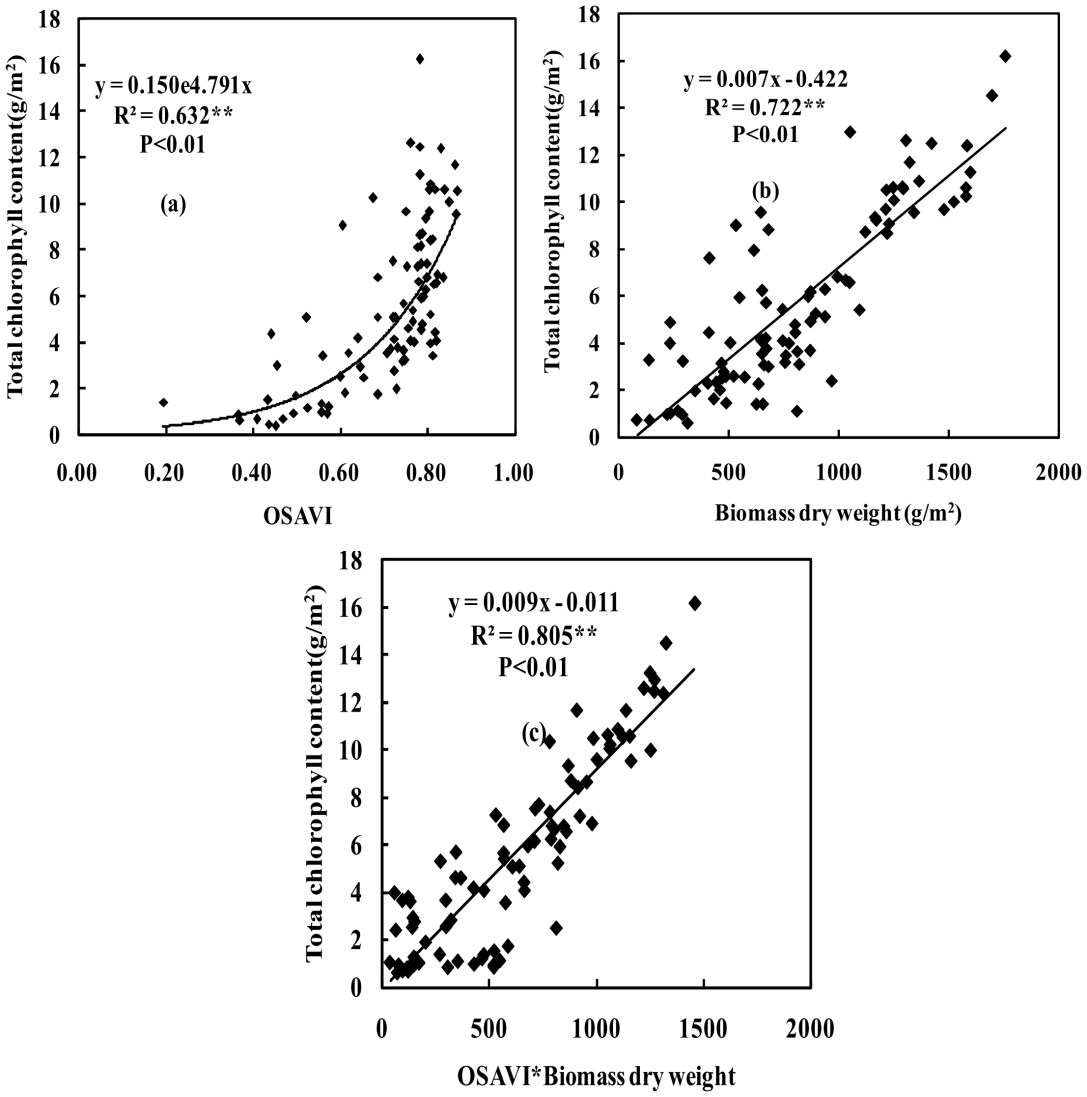
Quantitative relationship between OSAVI, biomass dry weight (BDW) and BDW×OSAVI and total chlorophyll content under the different nitrogen treatments for wheat (n = 90). Note: Same as above.

The total chlorophyll (Chl) content was correlated with OSAVI, with a coefficient of determination (R^2^) of 0.632. The results suggested that the total Chl content in the wheat canopy could be estimated using OSAVI (Figure 3a). Possible explanation could be the red edge absorption waveband (670 nm) is very sensitive to total Chl content [23]–[24], [26], [29]. Previous research also showed a good relationship between total Chl content and biomass [39], and biomass changes influenced the spectral reflectance in the near-infrared waveband (800 nm). Both red edge absorption waveband and near-infrared waveband are involved in OSAVI calculation. Therefore, variations in Chl content changes were detected by OSAVI.

Figure 3b shows that biomass dry weight and total chlorophyll content were more highly correlated than were the OSAVI and total Chl content, with an R^2^ value of 0.722. Linear regression was performed between biomass dry weight and total Chl content, and between biomass dry weight and total Chl content at the different growth stages. The results showed that the relationship between the OSAVI and total chlorophyll content exhibited similar results to that between OSAVI and N uptake vis-à-vis a lower correlation at earlier growth stages.

The OSAVI×biomass dry weight index could be used to estimate total Chl content changes, and it was a good indicator of total Chl content for wheat. It improved upon the results of the biomass dry weight and OSAVI alone for estimation of the total Chl content for wheat, and the R^2^ was 0.805 (Figure 3c). This result indicated that the OSAVI×biomass dry weight index improved upon the prediction accuracy for total Chl content.

Taken together, these results indicated that the OSAVI×biomass dry weight index (BDW) could be used to improve the estimation accuracy of LAI, nitrogen (N) uptake, and total chlorophyll (Chl) content, respectively. The most accurate estimation was gained for total Chl content, the worst for LAI, and the median for N uptake, among three agronomic parameters.

The results showed that biomass dry weight and LAI were highly correlated (R^2^ = 0.688, *P*<0.01) (Fig. 1b). Most of results indicated that the R^2^ and RMSE of the curves fitting was better than the linear fitting [40]–[43]. But sometimes, the linear fitting was better than the curves fitting, it may be a high related to measurement data and crops physiological mechanism. In this paper, we try to find the best fitting lines by using the curves fitting or the linear fitting, the result indicated that the equations of curves fitting is more than the equations of linear fitting, our results was consistent with previous researches [40]–[43]. Therefore, we justify the curves fitting was better than the linear fitting according to the R^2^ and RMSE of regression model. The results showed that biomass dry weight and LAI were highly correlated (Fig. 1b). It indicated that the size of biomass dry weight represented the size of LAI. It was mainly because LAI growth is synchronized with biomass dry weight according to the certain proportion. The biomass dry weight and nitrogen content were also highly correlated (Fig. 2b). It suggested that the appropriate nitrogen was used to increase the biomass dry weight accumulation. Because the nitrogen was applied for improving crops photosynthesis, and there was a good relationships between photosynthesis and biomass dry weight, thereby biomass dry weight accumulation was increased. Similarly, the appropriate total chlorophyll content could be used to increase crops photosynthesis and biomass dry weight accumulation, thus there was a high correlation between biomass dry weight and total chlorophyll content (Fig. 3b).

A straight linear relationships between biomass and LAI before heading stages, but this relationship between biomass and LAI have changed after heading stages. The rate of increase in the LAI is less than in the biomass (stem and spike weights increase more than leaf weight) before and after flowering. The LAI arrived at the highest values at flag leaf fully expanded, but the biomass dry weight (BDW) is still increased (it is mainly increased from spike weight). Therefore, a curvilinear relationship between wheat BDW and LAI should be better after heading stages. Metabolic physiology exists different before and after flowering. The wheat metabolic physiology was dominated by nitrogen and supplemented by carbon before flowering, canopy leaf nitrogen content change was relatively small, canopy leaf nitrogen content was accurately estimated by more accurately monitoring LAI. However, the wheat metabolic physiology was dominated by carbon and supplemented by nitrogen after flowering, the differences are significant in canopy leaf nitrogen content, nitrogen transfers from lower leaves to the upper leaves and spike in wheat. The nitrogen was not estimated accurately by monitoring LAI because of the lower leaves nitrogen was moved away by nitrogen transfer, resulting in a relative large error will be generated. If biomass factors are taken into account, thereby the transfer of nitrogen will also be included in the monitoring results, therefore it could be used to improve the nitrogen estimation accuracy. These results indicated that the biomass dry weight was highly related to LAI, nitrogen content and total chlorophyll content because biomass dry weight had a close relationship with LAI, nitrogen content and total chlorophyll content in crops physiological mechanism. These results also suggested that the biomass dry weight could be used to estimate the LAI, nitrogen content and total chlorophyll content for wheat. The results showed that a good relationships among the LAI, nitrogen content, total chlorophyll content and OSAVI×BDW (Figs. 1c, 2c and 3c). It provided a basis for BDW was replaced by spectral indices to establish new spectral indices.

### 3.4. New Spectral Indices

The new spectral index (OSAVI×BDW) had a good relationship with LAI, N uptake and total Chl content, but BDW was gained by destructive sampling, and required significant time investment for data acquisition. Thus, we attempted to build new spectral indices by replacing the BDW with others spectral indices. Tables 2, 3 and 4 showed that the new spectral indices: OSAVI×OSAVI, OSAVI×SIPI, OSAVI×CI_red edge_, OSAVI×CI_green model_ and OSAVI×EVI2, could be used to improve LAI, N uptake and total Chl content estimation accuracy. For LAI, the lowest and highest determination coefficient (R^2^) observed were OSAVI×SIPI and OSAVI×EVI2 with R^2^ values of 0.563 and 0.711, respectively (Table 2); similarly, the lowest and highest R^2^ were OSAVI×CI_green model_ and OSAVI×OSAVI, for which R^2^ were 0.542 and 0.785 for nitrogen uptake, respectively (Table 3); the lowest and highest R^2^ were OSAVI×CI_red edge_ and OSAVI×OSAVI, with R^2^ values of 0.652 and 0.846 for total Chl content, respectively (Table 4).

**Table 2:** Relationship between the new spectral indices and LAI under the different nitrogen treatments for wheat (n = 90).

Spectral indices	Simulated equations	Determination coefficient (R^2^)
OSAVI×OSAVI	y = 0.253ln(x)+0.221	0.634**
OSAVI×SIPI	y = 0.174ln(x)+0.507	0.563**
OSAVI×CI_red edge_	y = 3.756ln(x)+0.337	0.692**
OSAVI×CI_green model_	y = 3.534ln(x)+0.527	0.706**
OSAVI×EVI2	y = 0.269ln(x)+0.151	0.711**

Note: n = number of pairs of data. x represents spectral indices, y represents LAI.

Probability levels are indicated by n.s.,

* and ** for ‘not significant’, 0.05, and 0.01, respectively.

**Table 3:** Relationship between the new spectral indices and nitrogen uptake under the different nitrogen treatments for wheat (n = 90).

Spectral indices	Simulated equations	Determinationcoefficient (R^2^)
OSAVI×OSAVI	y = 0.246ln(x) − 0.095	0.542**
OSAVI×SIPI	y = 0.164ln(x) + 0.302	0.641**
OSAVI×CI_red edge_	y = 0.145x^1.331^	0.785**
OSAVI×CI_green model_	y = 0.203x^1.200^	0.776**
OSAVI×EVI2	y = 0.274ln(x) − 0.212	0.783**

Note: n = number of pairs of data. x represents spectral indices, y represents nitrogen uptake.

Probability levels are indicated by n.s.,

* and ** for ‘not significant’, 0.05, and 0.01, respectively.

**Table 4:** Relationship between the new spectral indices and total chlorophyll content under the different nitrogen treatments for wheat (n = 90).

Spectral indices	Simulated equations	Determinationcoefficient (R^2^)
OSAVI×OSAVI	y = 0.264x^0.400^	0.652**
OSAVI×SIPI	y = 0.504x^0.279^	0.683**
OSAVI×CI_red edge_	y = 0.137x^0.769^	0.846**
OSAVI×CI_green model_	y = 1.307x^0.742^	0.824**
OSAVI×EVI2	y = 0.208x^0.482^	0.798**

Note: n = number of pairs of data. x represents spectral indices, y represents total chlorophyll content.

Probability levels are indicated by n.s.,

* and ** for ‘not significant’, 0.05, and 0.01, respectively.

### 3.5. Validation Model and Comparison

To validate the model accuracy, we compared the predicted values (the predicted values were gained by the LAI, N uptake and total Chl content of regression equations in 2009) with the actual values (the actual values were gained by the LAI, N uptake and total Chl content field measurement data in 2010). A good correlation between the predicted values and the actual values was observed for the following indices: OSAVI, biomass dry weight, and OSAVI×biomass dry weight (BDW). The corresponding root mean square errors (RMSEs) were 1.42, 1.02, and 1.12 for LAI; 7.93 g/m^2^, 4.45 g/m^2^, and 4.23 g/m^2^ for nitrogen uptake; 3.42 g/m^2^, 1.85 g/m^2^, and 2.23 g/m^2^ for leaf chlorophyll content, respectively (Table 5). These data indicated that OSAVI×biomass dry weight could be used to improve the estimation accuracy of LAI, nitrogen uptake, and leaf chlorophyll content. The new spectral indices were proposed by replacing BDW with spectral indices, and then obtained OSAVI×OSAVI, OSAVI×SIPI, OSAVI×CI_red edge_, OSAVI×CI_green model_ and OSAVI×EVI2. The results showed that the new spectral indices were better than OSAVI alone for estimating LAI, nitrogen uptake and total chlorophyll content (Tables 2, 3, 4 and 5 and Figs. 1a, 2a and 3a). The results indicated that the products of spectral indices and OSAVI could be used to improve the LAI, N uptake and total Chl content estimation accuracy.

**Table 5:** The relationships the measured value and predicted value for LAI, nitrogen uptake and total chlorophyll content under the different nitrogen treatments for wheat.

LAI		Nitrogen uptake		Leaf chlorophyll content	
	RMSE (g/m^2^)		RMSE (g/m^2^)		RMSE (g/m^2^)
OSAVI	1.41	OSAVI	7.93	OSAVI	3.42
biomass dry weight	1.02	biomass dry weight	4.45	biomass dry weight	3.12
OSAVI×BDW	1.12	OSAVI×BDW	4.23	OSAVI×BDW	2.11
OSAVI×OSAVI	1.18	OSAVI×OSAVI	6.46	OSAVI×OSAVI	3.36
OSAVI×SIPI	1.36	OSAVI×SIPI	4.85	OSAVI×SIPI	3.02
OSAVI×CI_red edge_	0.98	OSAVI×CI_red edge_	3.98	OSAVI×CI_red edge_	0.65
OSAVI×CI_green model_	0.84	OSAVI×CI_green model_	4.12	OSAVI×CI_green model_	0.79
OSAVI×EVI2	0.78	OSAVI×EVI2	4.03	OSAVI×EVI2	1.02

## Discussion

Crop leaf area index (LAI), total chlorophyll (Chl) content and nitrogen (N) uptake was estimated using new spectral indices. The results showed that the new spectral indices could be used to improve the LAI, total Chl content and N uptake estimation accuracy. We used the OSAVI×biomass dry weight (BDW) index (the product of OSAVI and biomass dry weight) to improve the relationship between the LAI and spectral indices. The results indicated that the OSAVI×BDW index was better than the OSAVI at estimating wheat LAI. The OSAVI×BDW index was more sensitive to LAI due to the further decreased effects of soil at earlier growth stages. For example, the spectrometer was sensitive to the soil color and moisture, thus reducing the wheat canopy spectral information detection at earlier growth stages, and leading decreased sensitive to canopy spectral reflectance changes. The OSAVI×BDW ameliorated the saturation at later growth stages because of the addition of biomass dry weight, which was increased gradually with progression through the wheat growth stages. Overall, the saturation problem observed for the spectral indices was mainly influenced by the structure of the wheat canopy at later growth stages (see section 3.2). The OSAVI×BDW index was used to improve the estimation of nitrogen, and the results demonstrated that it was better than OSAVI alone for nitrogen assessment. The reason for this was similar to that explaining the reasonable estimation of LAI, the results were similar to those of previous research [14]–[19]. We proposed that the OSAVI×BDW index could be used to effectively improve the estimation accuracy of total Chl content indirectly, and the results demonstrated that it could accomplish the proposed tasks. Again, the reasons for such are similar to those explaining the reasonable estimation of N uptake.

The new spectral indices in Tables 2, 3 and 4 are derived from BDW×OSAVI. Biomass dry weight (BDW) was gained by destructive sampling, but required a greater time investment for data acquisition. To quickly obtain data and estimate leaf area index (LAI), total chlorophyll (Chl) content and nitrogen (N) uptake, we attempted to build new spectral indices by replacing BDW with other spectral indices. We obtained the OSAVI×OSAVI, OSAVI×SIPI, OSAVI×CI_red edge_, OSAVI×CI_green model_ and OSAVI×EVI2. The results suggested the new spectral indices were better than OSAVI alone for estimating LAI, total Chl content and N uptake (Tables 2, 3, 4 and 5 and Figs. 1a, 2a and 3a). The main reason is that the SIPI, CI_red edge_, CI_green model_ and EVI2 include the 800 nm band is sensitive to LAI changes. The products of OSAVI and SIPI, CI_red edge_, CI_green model_ and EVI2 will further improve the OSAVI sensitivity in LAI changes. Therefore, these new spectral indices could be used for improving the LAI. For N uptake, the products of OSAVI and SIPI, CI_red edge_, CI_green model_ and EVI2, making OSAVI increase the sensitive bands of the chlorophyll content changes (445 nm, 550 nm, 660 nm and 680 nm), these new spectral indices are sensitive to detect chlorophyll content changes. Thus, the N uptake estimation was improved by using the new spectral indices. For total Chl content, it was similar to those explained reasonable estimation of N uptake.

Taken together, the results indicated that it was feasible to use new methods to improve agronomic parameters (LAI, N uptake and total Chl content) assessment accuracy. This paper evaluated the estimation accuracy of agronomy parameters by multiplying the spectral indices in OSAVI, it showed the OSAVI is unnecessary and able to be replaced by others spectral indices. For example, if you want to better estimate total Chl content or N uptake, you could be selected to the product of two spectral indices are highly related to chlorophyll. Further, we used these indices to improve the accuracy of these predictions for all crop growth stages. In future studies, we will try to multiply two spectral indices that are highly related to LAI, total Chl content or N uptake to estimate agronomic parameters of different crops.

## Conclusions

Building upon previous studies, wheat leaf area index (LAI), nitrogen uptake, and total chlorophyll content were predicted using the products spectral indices and spectral indices methods, and the main results and conclusions follow. The results suggested that the LAI, nitrogen uptake and total Chl content could be accurately predicted using the products of OSAVI and biomass dry weight (OSAVI×biomass dry weight (BDW) index), the corresponding determination coefficient (R^2^) and root mean square errors (RMSEs) were 0.672 and 1.12, 0.754 and 4.23 g/m^2^, 0.805 and 2.11 g/m^2^, respectively. We obtained the new spectral indices by using spectral indices replace BDW. The relationships between LAI, nitrogen uptake and total Chl content and new spectral indices, OSAVI×EVI2, OSAVI×CI_red edge_ and OSAVI×CI_red edge_ had the highest R^2^ and the lowest RMSEs for LAI, nitrogen uptake and total Chl content, respectively, R^2^ and RMSEs were 0.711 and 0.78, 0.785 and 3.98 g/m^2^, 0.846 and 0.65 g/m^2^, respectively.
